# Dosage Strategy of Linezolid According to the Trough Concentration Target and Renal Function in Chinese Critically Ill Patients

**DOI:** 10.3389/fphar.2022.844567

**Published:** 2022-04-11

**Authors:** Fan Wu, Xiao-Shan Zhang, Ying Dai, Zi-Ye Zhou, Chun-Hong Zhang, Lu Han, Fang-Min Xu, Ye-Xuan Wang, Da-Wei Shi, Guan-Yang Lin, Xu-Ben Yu, Fang Chen

**Affiliations:** ^1^ Department of Pharmacy, The First Affiliated Hospital of Wenzhou Medical University, Wenzhou, China; ^2^ Department of Pharmacy, Wenzhou Medical University, Wenzhou, China; ^3^ Department of Pharmacy, The First Affiliated Hospital of Xiamen University, Xiamen, China

**Keywords:** linezolid, population pharmacokinetics, myelosuppression, dosage strategy, renal function

## Abstract

**Background:** Linezolid is associated with myelosuppression, which may cause failure in optimally treating bacterial infections. The study aimed to define the pharmacokinetic/toxicodynamic (PK/TD) threshold for critically ill patients and to identify a dosing strategy for critically ill patients with renal insufficiency.

**Methods:** The population pharmacokinetic (PK) model was developed using the NONMEM program. Logistic regression modeling was conducted to determine the toxicodynamic (TD) threshold of linezolid-induced myelosuppression. The dosing regimen was optimized based on the Monte Carlo simulation of the final model.

**Results:** PK analysis included 127 linezolid concentrations from 83 critically ill patients at a range of 0.25–21.61 mg/L. Creatinine clearance (CrCL) was identified as the only covariate of linezolid clearance that significantly explained interindividual variability. Thirty-four (40.97%) of the 83 patients developed linezolid-associated myelosuppression. Logistic regression analysis showed that the trough concentration (C_min_) was a significant predictor of myelosuppression in critically patients, and the threshold for C_min_ in predicting myelosuppression with 50% probability was 7.8 mg/L. The Kaplan–Meier plot revealed that the overall median time from the initiation of therapy to the development of myelosuppression was 12 days. Monte Carlo simulation indicated an empirical dose reduction to 600 mg every 24 h was optimal to balance the safety and efficacy in critically ill patients with CrCL of 30–60 ml/min, 450 mg every 24 h was the alternative for patients with CrCL <30 ml/min, and 600 mg every 12 h was recommended for patients with CrCL ≥60 ml/min.

**Conclusion:** Renal function plays a significant role in linezolid PKs for critically ill patients. A dose of 600 mg every 24 h was recommended for patients with CrCL <60 ml/min to minimize linezolid-induced myelosuppression.

## Introduction

Critical infection is recognized as an important determinant of outcome for patients in intensive care units (ICUs). Initial appropriate anti-infective therapy is associated with significantly improved clinical outcomes (Kollef et al., 1999; MacArthur et al., 2004; Kumar et al., 2006). However, achieving adequate exposure is challenging for critically ill patients because a variety of pathophysiological changes may significantly influence serum drug concentration.

Linezolid is predominantly metabolized through the oxidation of its morpholine ring to an inactive form by nonenzymatic oxidative reactions (Zurenko et al., 1996). According to the label sheet, the pharmacokinetics (PKs) of linezolid is insignificantly altered by patients’ age, gender, or by the presence of renal or hepatic insufficiency. Consequently, no dose adjustments are recommended for patients at any stage of renal dysfunction, including hemodialysis. However, the clearance (CL) of linezolid was found to increase by 50% during hemodialysis (Brier et al., 2003). Similarly, linezolid concentrations were significantly higher in patients with renal insufficiency than in those without (Wu et al., 2006; Matsumoto et al., 2009; Tsuji et al., 2011; Pea et al., 2012; Tsuji et al., 2013; Cossu et al., 2014; Matsumoto et al., 2014; Crass et al., 2019).

Myelosuppression, which can result in thrombocytopenia and anemia, is the most frequent adverse effect in patients receiving linezolid. Thrombocytopenia exhibits the highest incidence, occurring in >30% of patients undergoing linezolid therapy (Lin et al., 2006; Wu et al., 2006; Ikuta et al., 2011; Takahashi et al., 2011; Nukui et al., 2013; Dong et al., 2014; Hirano et al., 2014). The incidence of anemia among such patients is 2.8–47.3% (Senneville et al., 2004; Lin et al., 2006; Takahashi et al., 2011; Pea et al., 2012). Risk factors for the development of thrombocytopenia during linezolid therapy include renal insufficiency (Lin et al., 2006; Ikuta et al., 2011; Takahashi et al., 2011; Hirano et al., 2014), long-term linezolid administration (Gerson et al., 2002; Takahashi et al., 2011), low baseline platelet count (Niwa et al., 2009; Cattaneo et al., 2013; Dong et al., 2014), and low body weight (Niwa et al., 2009; Dong et al., 2014). Similarly, the risk factors for linezolid-induced anemia include renal insufficiency, age, and low baseline hemoglobin count (Wu et al., 2006; Tsuji et al., 2011). In addition, renal impairment has been identified as a significant factor for elevated linezolid exposure, which was the main cause of myelosuppression (Morata et al., 2016; Pea et al., 2017). A recent study pointed out that the linezolid dosing in renal impairment patients should be reappraised to improve safety (Crass et al., 2019). However, this study was conducted in a population that did not include critically ill patients. Besides, the recommended dosage in patients with renal impairment only achieved approximately 65% of the therapeutic target. Therefore, a dose modification and more frequent monitoring of linezolid exposure are necessary for critically ill patients.

The present study aimed to define the therapeutic range based on linezolid trough concentration in critically ill patients. The application of drug therapeutic monitoring based on single blood sampling is much more convenient in clinics than monitoring AUC. We also determined a dosage modification for critically ill patients with renal impairment.

## Materials and Methods

### Patients and Ethics

This was a retrospective, observational study of hospitalized critically ill patients receiving linezolid for confirmed or suspected multiresistant Gram-positive bacterial infections from January 2018 to December 2019 at the First Affiliated Hospital of Wenzhou Medical University. The inclusion criteria were as follows: 1) patients receiving intensive care aged ≥18 years with confirmed or suspected Gram-positive bacterial infections; 2) patients who received intravenous linezolid for at least 3 days; and 3) patients for whom at least one steady-state concentration of linezolid was collected. The exclusion criteria were as follows: 1) patients who received renal replacement therapy or extracorporeal membrane oxygenation; 2) patients who died within 24 h after being treated with linezolid; 3) patients with baseline PLT <75 × 10^9^ cells//L; 4) patients with baseline hemoglobin <6.8 g/dl for males or <6 g/dl for females; 5) patients with baseline absolute neutrophil count <500 cells/μl; and 6) patients with baseline TBIL > 5 times the upper limit of normal. The baseline was defined at the initiation of linezolid therapy.

The study was designed in accordance with legal requirements and the Declaration of Helsinki and was approved by the Ethical Committees of the First Affiliated Hospital of Wenzhou Medical University, China [(2021)034], registered at the Chinese Clinical Trial Registry (ChiCTR2100047882). The informed consent was free passed by the Ethics Committee in Clinical Research (ECCR) of the First Affiliated Hospital of Wenzhou Medical University.

### Pharmacokinetic Sampling

Routine therapeutic drug monitoring (TDM) data of linezolid were retrospectively obtained from a database maintained at the Department of Pharmacy. The decision to administer linezolid and its dosing regimens (dose amount, dosing interval, duration of intravenous administration, and duration of therapy) were made by the attending physician. An opportunistic sampling strategy was performed when a steady-state concentration of linezolid has been achieved (at least 3 days from the start of treatment). Most of the TDM samples collected were peak or trough concentrations. The concentration–time profile of linezolid for the included patients is shown in Supplementary Figure S1. The exact time of linezolid treatment and TDM sampling were able to be indexed.

Plasma samples were separated by centrifugation for 5 min at 15,000 rpm and were determined within 24 h after sampling. Quantification of the plasma concentration of linezolid was performed using a validated high-performance liquid chromatography–tandem mass spectrometry (LC-MS/MS) assay (Phillips et al., 2001). Intra- and inter-day assay coefficients of variation were <10%, and the lower limit of quantification was 0.1 mg/L. Individual laboratory parameters and demographic data of patients, including gender, age, height, body weight (WT), white blood cell count (WBC), hemoglobin (Hb), platelet count (PLT), total bilirubin (TBIL), serum albumin (ALB), alanine aminotransferase (ALT), aspartate aminotransferase (AST), and serum creatinine (SCr), were collected. CrCL was calculated using the Cockcroft–Gault equation. Data organization and visualization were performed using R (version 3.6.1) and R Studio (version 1.3.1093).

### Hematological Toxicity Analysis of Linezolid

Myelosuppression was defined as follows: 1) thrombocytopenia: PLT <125 × 10^9^ cells/L and a decrease of ≥25% PLT in comparison with the baseline levels; 2) anemia: a reduction of ≥25% of Hb compared with the baseline. The baseline levels were defined as the hematological parameters at the initiation of linezolid therapy.

### Population Pharmacokinetic Modeling of Linezolid

Population PK analysis was performed using the nonlinear mixed effects modeling program NONMEM (version 7.4, Icon Development Solutions, Ellicott City, MD, United States) and Pirana (version 2.9.7). R (version 3.6.0) and Xpose (version 4.3.2) software packages were applied to generate diagnostic plots. The first-order conditional estimation method with inter- and intra-subject variability was used throughout the model development procedure. One- and two-compartment structural models with first-order elimination were explored for the linezolid plasma concentration–time profiles. Between-subject variability (BSV) was modeled using exponential function. Residual variability was assessed using additive, proportional, and combined (additive plus proportional) error models. The base model was selected based on the visual inspection of diagnostic plots and various goodness-of-fit criteria, including precision and plausibility of parameter estimation, improvement of the objective function value (OFV), Akaike information criteria (AIC), and Bayesian information criterion (BIC).

Covariates were included using a stepwise forward selection process with a threshold decrease in OFV of 3.84 (*p* < 0.05, 1 degree of freedom [df]) until no further decrease in OFV was observed. All of the significant covariates were then incorporated into the basic model to construct a full model. In backward elimination, the covariate was retained in the final model with a threshold increase in OFV of 6.63 (*p* < 0.01, 1 df); otherwise, it was eliminated from the model. The additional criteria for retaining the covariate in the final model were a decrease in the unexplained BSV and an increase in PK parameter estimate precision.

For internal validation of the final model, goodness-of-fit plots, including observed concentrations (DV) versus individual prediction (IPRED), DV versus population prediction (PRED), conditional weighted residuals (CWRES) versus PRED, and CWRES versus time after last dose (TAD), were performed. A nonparametric bootstrap procedure was conducted to assess the performance and stability of the final model (Ette and Onyiah, 2002; Ette et al., 2003). Random sampling with replacement was utilized to generate 1,000 replicate datasets using the individual as the sampling unit. The median and 95% confidence intervals (CIs) of the resulting parameters were calculated and compared with the final parameter estimates obtained using the NONMEM program. To evaluate the predictive performance, the statistics of the observed and simulated time–concentration profiles were compared using prediction- and variability-corrected visual predictive check (pvcVPC) (Bergstrand et al., 2011). The dataset was simulated 1,000 times using the $SIMULATION block in NONMEM^®^ for pvcVPC. The 90% CIs for the 5th, 50th, and 95th percentiles of the simulated concentrations were calculated, plotted against time after the last dose, and compared with the observed concentrations.

### Monte Carlo Simulation

Monte Carlo simulations were performed using the final model to identify the pragmatic dose adjustment of linezolid. Simulations were performed with the covariate sets from individual patients included in model building, serving as a template for 1,000 simulated subjects. The probability of target attainment (PTA) of the C_min_ target was calculated for each dose (daily doses of 300–1,800 mg/day with increments between 150 and 300 mg) among simulated subjects with CrCL values of <30 ml/min, 30–59 ml/min, 60–89 ml/min, and ≥90 ml/min.

### Statistical Analyses

Statistical analyses were performed using SPSS version 21.0 (IBM Corp). All study variables were summarized by descriptive statistics. The C_min_ and AUC_0-24_ at steady state of each patient were predicted via the maximum a posteriori probability (MAP) Bayesian function of NONMEM using the final PK model as the Bayesian prior. AUC_0-24_ was calculated by the linear-log trapezoidal rule using the concentrations at continuous time (10 min-interval) predicted via Bayesian estimation. Specifically, the linear trapezoidal approach was used during the ascending phase and the log-linear method was used during the descending phase. Logistic regression modeling for the toxicity of linezolid was conducted to determine whether the trough concentration or AUC_0-24_ of linezolid was a significant predictor of myelosuppression during treatment. The time from the initiation of linezolid treatment to the development of myelosuppression was estimated using the Kaplan–Meier curve analysis. All statistical tests were two-sided. *p* values <0.05 were considered statistically significant.

## Results

### Baseline Characteristics of Patients

In total, 83 critically ill patients with a mean ± S.D. age of 60.57 ± 14.64 years and body weight of 64.11 ± 11.21 kg were included in the present study. The demographic data of the included patients are summarized in Table 1. The overall rate of myelosuppression occurrence, including thrombocytopenia and anemia, in this study was 40.97%. The incidence rates of thrombocytopenia and anemia were 36.14 and 16.87%, respectively. The median daily dose of linezolid selected by the physician was 20.17 mg/kg/d, and most patients received the drug every 12 h.

**TABLE 1:** Baseline characteristics of patients included for pharmacokinetic analysis set or hematological toxicity analysis set.

Characteristic	Value^a^
Age (years)	62 [16, 99]
Sex	
Male	54 (65.06%)
Female	29 (34.94%)
Height (cm)	165 [143, 181]
Total body weight (kg)	64 [40, 110]
Daily dose (mg/kg/d)	20.17 [11.33, 33.27]
Treatment duration (days)	8 [4, 58]
MRSA microbiology: positive	15 (16.85%)
Myelosuppression	34 (40.97%)
Thrombocytopenia (n, %)	30 (36.14%)
Anemia (n, %)	14 (16.87%)
Clinical data	
Hemoglobin (g/L)	99 [67, 170]
Platelet (⨉10^9^/L)	232 [64, 658]
White cell count (⨉10^9^/L)	8.68 [3.11, 87.86]
Total bilirubin (μmol/L)	10 [2.5, 87]
AST (U/L)	29 [10, 5,742]
ALT (U/L)	32 [1, 2,449]
ALB (g/L)	30 [12, 42]
Serum creatinine (μmol/L)	81 [33, 687]
CrCL (ml/min)	65.38 [9.99, 195.60]

Abbreviations; AST, aspartic transaminase; ALT, alanine transaminase; ALB, serum albumin; CrCL, estimated creatinine clearance (CrCL) calculated using the Cockcroft–Gault equation.

a Values are No. (%) or median [minimum, maximum].

### Population Pharmacokinetic Analysis

A total of 127 linezolid concentrations derived from 83 patients at a range of 0.25–21.61 mg/L were obtained for population PK modeling. The PK characteristics of linezolid illustrated by a 1-compartment model with linear elimination showed the best fit of the observed concentration–time data based on reduction in OFV and residual variability. The intra-individual variability for V was fixed as 0 because it was very small (*ŋ*<0.0025); it might be because of the limited data of peak concentrations. The proportional error model was used to evaluate the residual variability. Parameter estimates and diagnostic plots of the base model are provided in Supplementary Table S1 and Supplementary Figure S2. Covariate model building identified CrCL as the only covariate on linezolid CL that significantly explained interindividual variability. The final PK model is represented as follows:

$$CL\left(L/h\right)=3.66+2.18\times \frac{CrCL}{65},$$ (1)

V(L)=54, (2)

where CL is the individual clearance, V is the individual volume of distribution, and CrCL is the estimated creatinine clearance. The parameter estimates of the final model are displayed in Table 2.

**TABLE 2:** Population pharmacokinetic parameter estimates from the final model.

Parameter	Estimate (%)	RSE (%)	Shrinkage (%)
Fixed effects			
TVCL [L/h]	3.66	15	
CrCL on CL	2.18	21	
TVV [L]	54	17	
Between-subject variability (BSV)^a^			
BSV_CL [%CV]	36.30	10	3
Residual variability (RV)			
Proportional error [%CV]	19.05	19	24

a BSV calculated as $\sqrt{e^{\omega^{2}}-1}$ .

The mean ± SD individual empirical Bayesian estimate of CL was 6.35 ± 2.32 L/h, across all patients with V estimated at 54 L. Furthermore, dividing the included patients into the group with linezolid-induced myelosuppression and the group without, the estimated CL was significantly lower in patients with linezolid-induced myelosuppression compared with that in the patients without (4.91 ± 1.51 L/h vs. 7.57 ± 2.35 L/h, *p* = 0.027).

**FIGURE 1:**
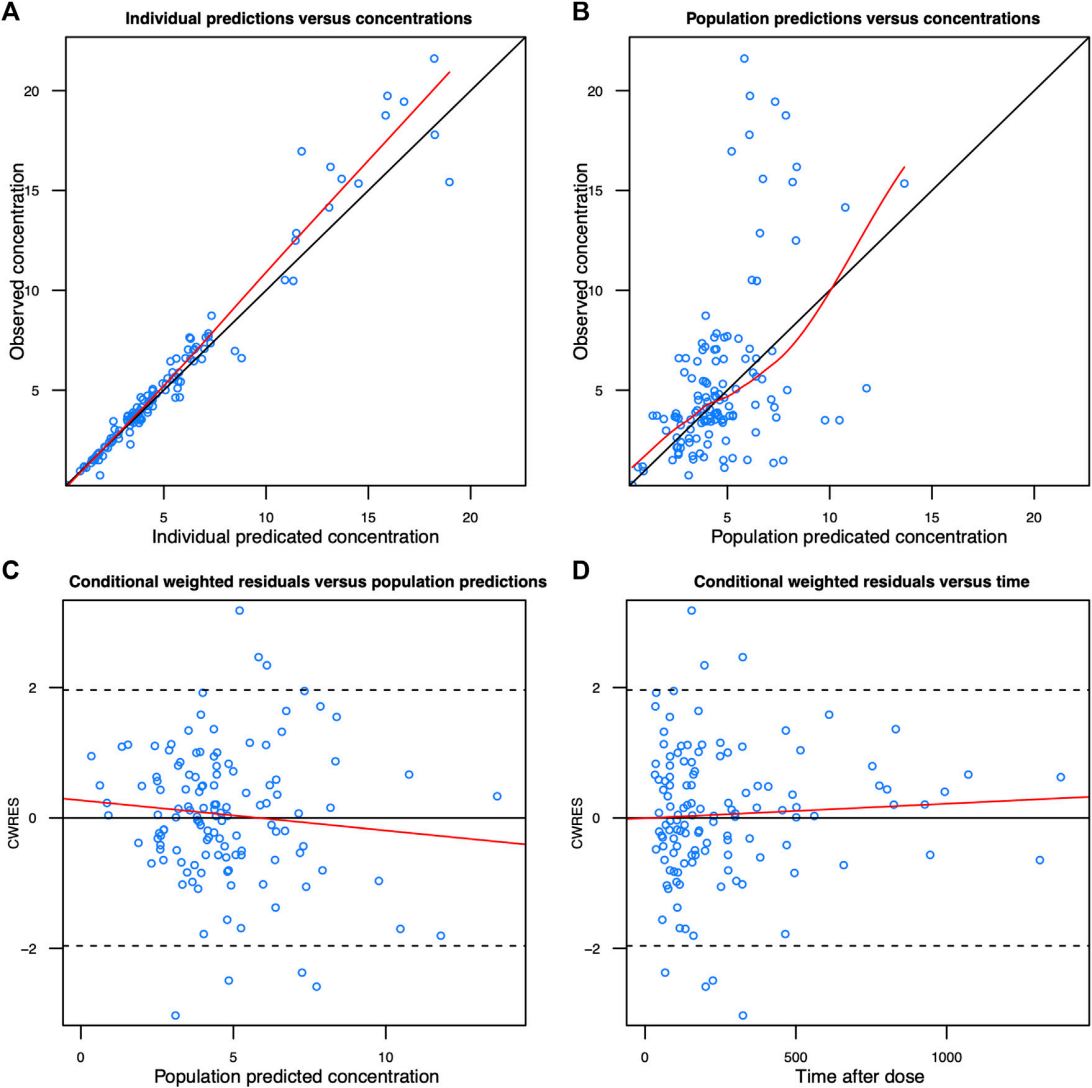
Diagnostic goodness-of-fit plots of the final model. **(A)** Observed concentration (DV) vs. individual predicted concentration (IPRED); **(B)** DV vs. population-predicted concentration (PRED); **(C)** conditional weighted residuals (CWRES) vs. PRED; and **(D)** CWRES vs. time. The red lines in the upper panel represent loess smooth lines and linear fit lines, respectively.

The diagnostic goodness-of-fit plots of the final model are shown in Figure 1. The CWRES versus PRED of the final model showed a stochastic distribution around zero, and most residuals were within an acceptable range (−2 to 2). The median with 95% CI parameter estimates obtained from a 1,000-run bootstrap analysis is given in Supplementary Table S2. The parameter estimates of the final PK model lay within the 95% CIs from the nonparametric bootstrap procedure, and the biases between the final model estimates and the bootstrapped median parameter estimates were within ±10% for all parameters, which demonstrated the good stability of the final model. The pvcVPC of concentrations versus time after the last dose reflected a good fit between the observations and simulations (Supplementary Figure S3). Overall, the linezolid population PK model evaluation results revealed that the final model provided an adequate description of the data and a good prediction of individual PK parameters.

### Linezolid Therapeutic Target Analyses

A strong correlation (r2 = 0.9969) was found between the C_min_ and AUC0-24. The linear regression line was AUC0-24 = 26.354×C_min_+91.607. The ratio of the AUC to MIC was determined to be the most important PK/PD index for linezolid against strains of *S. aureus* and strains of *S. pneumoniae* with varying antibacterial susceptibilities, and the antibacterial activity of linezolid may be maximized when AUC0-24/MIC ratio ≥80, which was derived from a study conducted in critically ill patients (Rayner et al., 2003). Given the MIC90 of linezolid for most staphylococci is 2 mg/L (Jones et al., 2007), to achieve the PK/PD index (AUC0-24/MIC ≥80) for the efficacy of linezolid, an AUC0-24 of 160 mg h/L was required when the MIC was 2 mg/L. Using the linear regression equation, the C_min_ for AUC0-24 = 160 mg h/L was 2.6 mg/L. Thus, the C_min_ required for sufficient efficacy was ≥2.6 mg/L.

**FIGURE 2:**
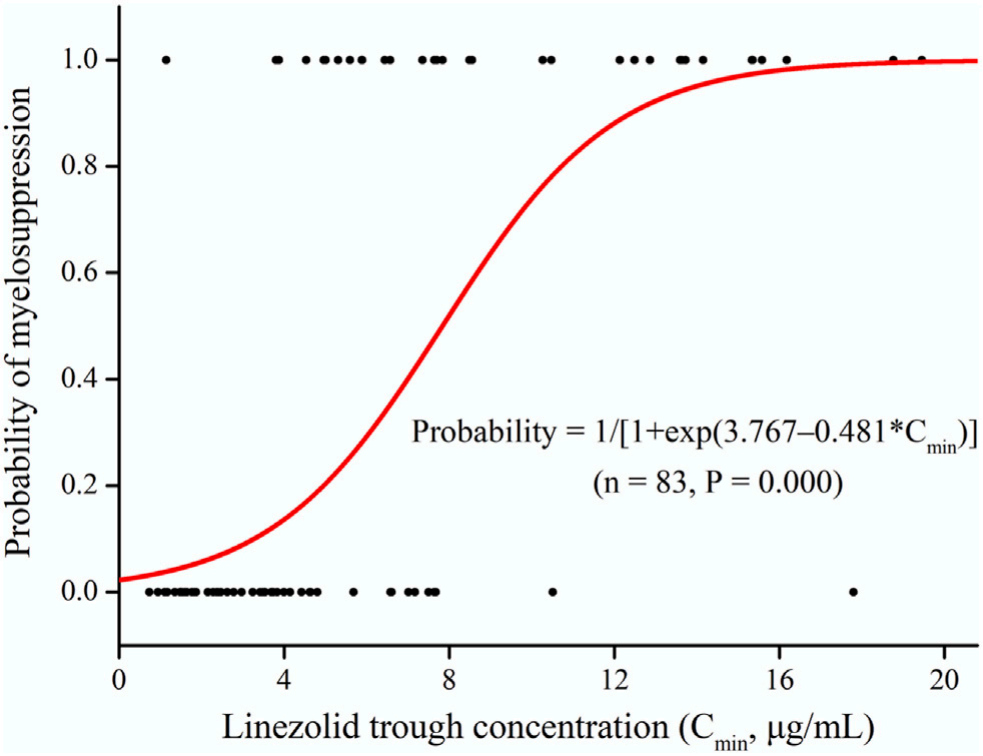
Linezolid trough plasma concentrations (C_min_) and logistic regression model for myelosuppression (absence, 0; presence, 1).

**FIGURE 3:**
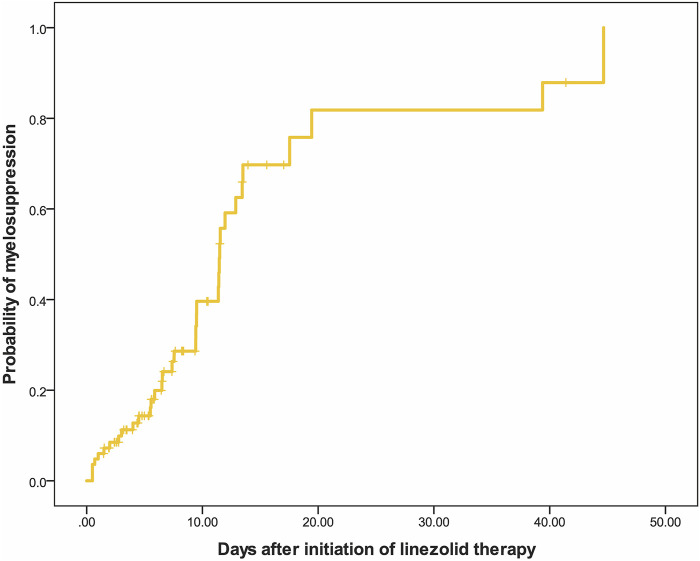
Kaplan–Meier plot showing the time from the initiation of linezolid therapy to the development of myelosuppression (n = 83).

Linezolid-induced myelosuppression was observed in 34 (40.97%) of the 83 patients. Figure 2 shows the relationship between the C_min_ and the myelosuppression (absence, 0; presence, 1). The C_min_ of linezolid was a significant predictor of myelosuppression in critically patients according to the following equation: probability of myelosuppression = 1/[1 + exp (3.767–0.481*C_min_)]. The threshold for the C_min_ of linezolid that caused myelosuppression with 50% probability was 7.8 mg/L. In general, the target C_min_ range based on the aforementioned results was considered to be 2.6–7.8 mg/L. In addition, the Kaplan–Meier plot revealed that the overall median time from the initiation of therapy to the development of myelosuppression was 12 days (Figure 3).

### Monte Carlo Simulation

**FIGURE 4:**
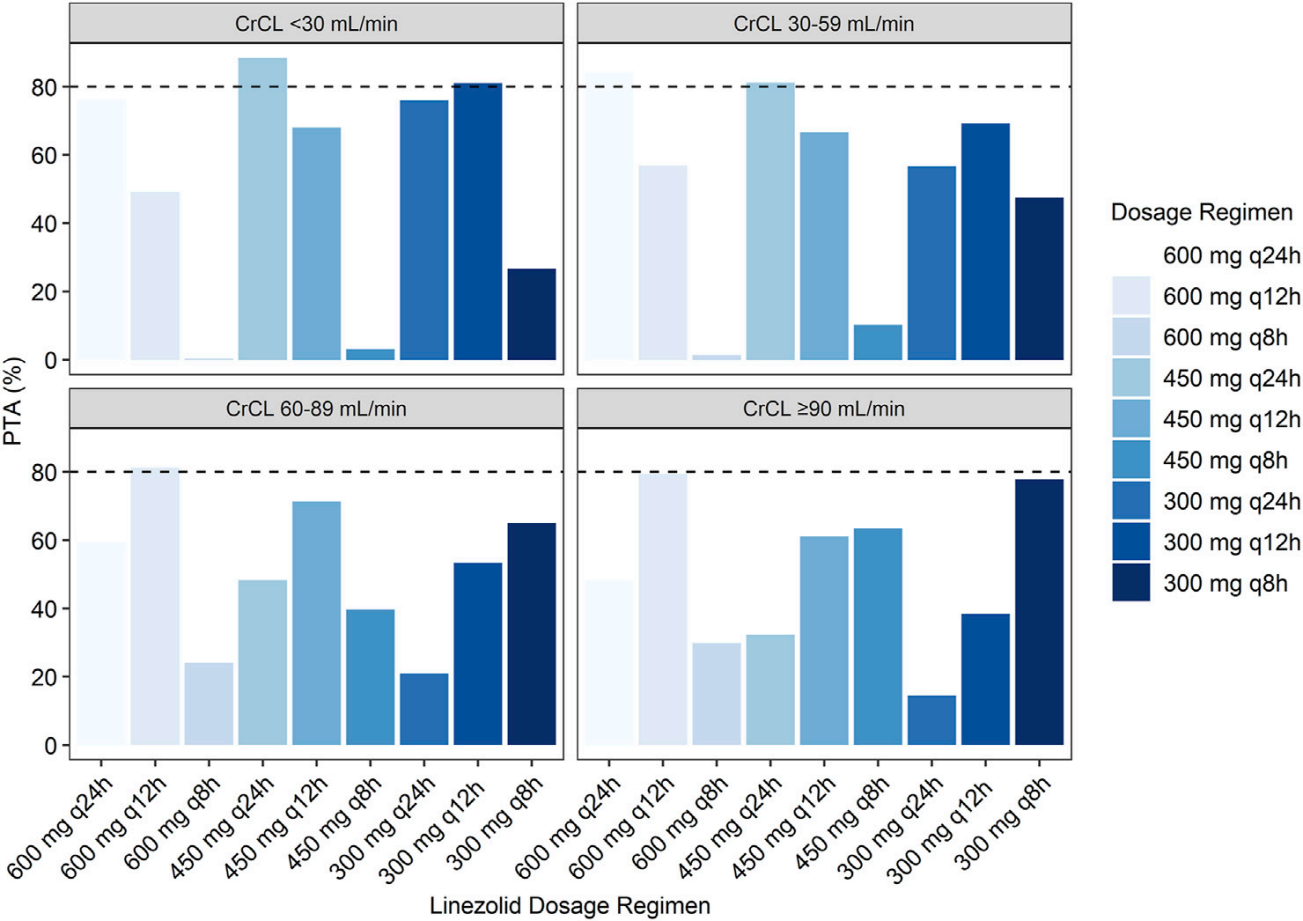
Simulated probability of achieving target attainment of linezolid (C_min_: 2.6–7.8 mg/L) stratified by renal function.

The simulated PTA of linezolid therapeutic range (C_min_ 2.6–7.8 mg/L) with various dosage regimens in patients with various CrCLs were quantified in Table 3 and Figure 4. The target for PTA was defined as > 80%. The low probability of attaining therapeutic C_min_ values in patients with renal impairment (CrCL <60 ml/min) with a standard linezolid dose of 600 mg every 12 h is primarily due to an increase in the probability of attaining supratherapeutic C_min_ (>7.8 mg/L). An empirical dose reduction to 600 mg every 24 h was the optimal to balance safety and efficacy in patients with CrCL of 30–59 ml/min, whereas 450 mg every 24 h was the alternative for patients with CrCL <30 ml/min.

**TABLE 3:** Simulated probability of attaining linezolid trough concentrations associated with efficacy and toxicity stratified by renal function.

Linezolid dosage regimen	%Probability			
	CrCL <30 ml/min	CrCL 30–59 ml/min	CrCL 60–89 ml/min	CrCL ≥90 ml/min
600 mg q24h	76.30	**84.17**	59.47	48.30
600 mg q12h	49.13	56.90	**81.20**	**79.33**
600 mg q8h	0.33	1.43	24.13	29.8
450 mg q24h	**88.40**	81.17	48.30	32.30
450 mg q12h	67.97	66.57	71.33	61.10
450 mg q8h	3.13	10.27	39.67	63.43
300 mg q24h	76	56.67	20.93	14.4
300 mg q12h	81.10	69.27	53.33	38.40
300 mg q8h	26.67	47.50	65.07	77.80

Abbreviations; CrCL, estimated creatinine clearance (CrCL) calculated using the Cockcroft–Gault equation.

The bold values are the values with the highest PTA of the dosage regimen stratified by renal function.

## Discussion

This is the first study focused on critically ill patients aimed at drawing the therapeutic target of linezolid as well as dosage modification for critically ill patients with renal impairment. The therapeutic range of C_min_ was considered to be 2.6–7.8 mg/L to balance the efficacy and safety of linezolid, and linezolid treatment for ≥12 days was associated with the risk of myelosuppression. This study also developed a population PK model, which showed a significant relationship between CrCL and the total clearance of linezolid, and dose reduction to 600 mg every 24 h was the alternative for critically ill patients with CrCL of 30–59 ml/min, whereas 450 mg every 24 h was the alternative for patients with CrCL <30 ml/min.

The results of the PK analysis indicated that the one-compartment model with first-order elimination along with CrCL as a significant covariate on CL was optimal for the PK data modeling. The typical value of CL in the final PK model of this study was 6.35 L/h, which is consistent with other PK studies of linezolid performed in critically ill patients (Taubert et al., 2016; Soraluce et al., 2020). A comparison of our estimates of PK parameters with those reported in the literature is presented in Supplementary Table S3 (Whitehouse et al., 2005; Plock et al., 2007; Abe et al., 2009; Keel et al., 2011; Sasaki et al., 2011; Tsuji et al., 2013; Luque et al., 2014; Matsumoto et al., 2014; Taubert et al., 2016; Töpper et al., 2016; Zhang et al., 2016; Tsuji et al., 2017; Crass et al., 2019; Garcia-Prats et al., 2019; Li et al., 2019; Wang et al., 2019; Xie et al., 2019; Soraluce et al., 2020). According to the label of linezolid, its clearance occurs by both renal and hepatic mechanisms (approximately 30 and 65%, respectively). Thus, the covariates associated with renal and hepatic functionality were analyzed in the population PK analysis. However, only CrCL was included in the final model. Despite using the available bilirubin and transaminases data as candidate covariates, none of them improved the PK model. It might be due to the lack of reliable, economical, and untroublesome parameters of liver function that account for the drug clearance (Watkins et al., 2014; Wicha et al., 2017). Linezolid is currently administered at a fixed dose of 600 mg every 12 h despite the high between-subject variability in exposure and reduced CL in patients with renal insufficiency. It has been reported that renal impairment and end-stage renal disease have been associated with an increased risk of developing thrombocytopenia in patients receiving linezolid (Wu et al., 2006; Matsumoto et al., 2009; Takahashi et al., 2011; Hanai et al., 2016; Rabon et al., 2018). In addition, a clear exposure–toxicity relationship has been identified for myelosuppression (Matsumoto et al., 2010; Tsuji et al., 2011; Cattaneo et al., 2013; Dong et al., 2014). Thus, it is likely that increased linezolid exposure is the underlying reason for the higher risk of linezolid-related toxicity in patients with renal impairment. In addition, while renal function also plays a critical role in the clearance of the major metabolites of linezolid (Souza et al., 2020), linezolid and its major metabolites share a chemical feature (aniline functional group) which medicinal chemistry has demonstrated to be a risk factor for myelosuppression (Crivori et al., 2011; Stepan et al., 2011).

Previous studies have assessed the association between the C_min_, AUC0-24 of linezolid and thrombocytopenia. The first report was published by Matsumoto et al. in 2010 (Matsumoto et al., 2010), who found that patients developing thrombocytopenia had linezolid trough concentrations ranging from 14.4 to 35.6 mg/L, and they developed a logit model equation identifying a trough concentration of 22.1 mg/L as the upper threshold for risk of thrombocytopenia (Hiraki et al., 2012). However, this proposed target was challenged by the findings from Pea et al. and Matsumoto et al. who identified a much lower threshold of trough concentration (6.5 and 8.1 mg/L, respectively) associated with 50% probability of risking thrombocytopenia (Pea et al., 2012; Matsumoto et al., 2014). The present study showed that the C_min_ threshold of linezolid that caused myelosuppression with 50% probability was 7.8 mg/L in critically ill patients. Using the linear regression equation, the AUC0-24 for C_min_ of 7.8 mg/L was estimated to be 297.2 mg h/L, which was consistent with previous findings (Pea et al., 2012; Cattaneo et al., 2013; Nukui et al., 2013). In addition, we showed that administration of linezolid for ≥12 days was associated with a high risk of myelosuppression. Therefore, critically ill patients receiving linezolid for ≥12 days should be monitored carefully. Individualized dosing and monitoring of linezolid trough concentration could potentially help overcome the limitation of linezolid toxicity, especially in renal insufficiency patients. The previous studies suggested that a CrCL of <30 ml/min should be used as a threshold for predicting the risk of linezolid-induced thrombocytopenia (Sasaki et al., 2011; Hirano et al., 2014), whereas YuKi et al. pointed out that patients with CrCL of <60 ml/min and those on hemodialysis are at high risk of thrombocytopenia, which is consistent with our result (Hanai et al., 2016). However, the dose adjustment recommendation was not provided by the study. In the present study, according to the simulated probability of attaining linezolid target trough concentrations, a dose reduction to 600 mg every 24 h was the alternative for patients with CrCL of 30–59 ml/min, whereas 450 mg every 24 h was the alternative for patients with CrCL <30 ml/min. However, it should be mentioned that dosage adjusted according to CrCL could not always achieve the target trough concentration in all patients because inter-individual variability still exists. In addition, it should also be noted that the dosage regimen should be subsequently adjusted following the change in the renal function, especially for critically ill patients whose initial renal impairment is caused by severe infection, because their renal impairment will soon be improved after alleviating the severe infection. TDM has been suggested by some authors to optimize linezolid therapy in critically ill patients (Pea et al., 2010; Cattaneo et al., 2013). Therefore, the combination of using the recommended initial dose and subsequently adjusting the dose guided by TDM could enable effective linezolid therapies while avoiding adverse events.

There are some limitations to this study. First, PK analysis was performed using the data obtained from linezolid TDM retrospectively, even though the dosing time and sampling time were precisely recorded. Second, we did not investigate the effects of concomitant medications that are capable of causing myelosuppression. Third, the effect of renal replacement therapy on linezolid clearance was not clarified in the study, since patients who had renal replacement therapy were excluded from this study. Fourth, the distribution of plasma concentrations collected in this study was mainly trough concentrations, and only a small part of peak concentrations was included. Thus, we admitted that the estimated apparent volume and prediction of peak concentrations in the population PK model may not fit well because of the limited data of peak concentrations.

In conclusion, the population PK analysis revealed that renal function significantly affects the PKs of linezolid. The therapeutic target for linezolid was considered to be C_min_ of 2.6–7.8 mg/L to minimize linezolid-induced myelosuppression while maintaining treatment efficacy in critically ill patients. Furthermore, a simulation based on the constructed PK model suggested a reduced dose of 600 mg every 24 h was recommended for patients with CrCL of 30–59 ml/min, whereas 450 mg every 24 h was the alternative for patients with CrCL <30 ml/min. Given the high between-subject variability of linezolid PKs, TDM is necessary to ensure optimal therapy across patient population.

## Data Availability

The original contributions presented in the study are included in the article/Supplementary Material, further inquiries can be directed to the corresponding authors.
