# Supplementary material for: Dosage Strategy of Linezolid According to the Trough Concentration Target and Renal Function in Chinese Critically Ill Patients
Source: Front Pharmacol. 2022 Apr 11;13:844567. doi: 10.3389/fphar.2022.844567 (PMC9035989; doi:10.3389/fphar.2022.844567)
Supplement: Supplementary file 1 [file DataSheet1.ZIP › Supplementary materials/Control stream.docx]

**Control file of Final mode**l

;1-CMT linear elim

$PROBLEM LZD 1 cmt, CL,V linear elim

$INPUT ID PID DAT2=DROP TIME DAY TAD DV AMT CMT CTIME RTE EVID MDV SEX AGE BH BW TBP ANEMIA WBC Hb PLT TBIL ALB ALT AST Cr CRCL

$DATA ppp.csv

$SUBROUTINES ADVAN1 TRANS2

$PK

TVCL=THETA(1)+THETA(3)*CRCL/65.38

CL=TVCL*EXP(ETA(1))

V=THETA(2)

S1=V

$ERROR

IPRED=F

IRES=DV-IPRED

DEL=0

IF(DV.EQ.0) DEL=1

IWRES=(1-DEL)*IRES/(DV+DEL)

Y=F+F*EPS(1)

$THETA

10;CL

30;V

1;CL_CRCL

$OMEGA

(0.1);IIV on CL

$SIGMA

(0.1);Pro_Err

$EST METHOD=1 INTER MAXEVAL=9999 PRINT=5 NOABORT POSTHOC

$COV PRINT=E

$TABLE ID TIME TAD DV MDV EVID PRED IPRED IWRES CWRES NOPRINT ONEHEADER FILE=sdtab001a

$TABLE ID TAD CL V ETA1 NOPRINT ONEHEADER FILE=patab001a

$TABLE ID AGE BH BW WBC Hb PLT TBIL ALB ALT AST Cr CRCL NOPRINT ONEHEADER FILE=cotab001a

$TABLE ID SEX TBP ANEMIA NOPRINT ONEHEADER FILE=catab001a

**Control file of simulation**

;1-CMT linear elim

$PROBLEM LZD 1 cmt, CL,V linear elim

$INPUT ID PID DAT2=DROP TIME DAY TAD DV AMT CMT CTIME RTE EVID MDV SEX AGE BH BW TBP ANEMIA WBC Hb PLT TBIL ALB ALT AST Cr CRCL

$DATA ppp.csv

$SUBROUTINES ADVAN1 TRANS2

$PK

TVCL=THETA(1)+THETA(3)*CRCL/65.38

CL=TVCL*EXP(ETA(1))

V=THETA(2)

S1=V

$ERROR

IPRED=F

IRES=DV-IPRED

DEL=0

IF(DV.EQ.0) DEL=1

IWRES=(1-DEL)*IRES/(DV+DEL)

Y=F+F*EPS(1)

$THETA

3.66;CL

54;V

2.18;CL_CRCL

$OMEGA

0.132;IIV on CL

$SIGMA

0.0363;Pro_Err

$SIMULATION (123456) ONLYSIM SUBPROBLEM=1000

$TABLE ID TIME TAD DV MDV PRED IPRED NOPRINT ONEHEADER FILE=.fit
