# Supplementary material for: Dosage Strategy of Linezolid According to the Trough Concentration Target and Renal Function in Chinese Critically Ill Patients
Source: Front Pharmacol. 2022 Apr 11;13:844567. doi: 10.3389/fphar.2022.844567 (PMC9035989; doi:10.3389/fphar.2022.844567)
Supplement: Supplementary file 1 [file DataSheet1.ZIP › Supplementary materials/Supplementary tables and figures.docx]

**Table S1 Population pharmacokinetic parameter estimates from the base model**

| **Parameter** | **Estimate** | **RSE** | **shrinkage** |
| --- | --- | --- | --- |
| **Fixed Effects** |  |  |  |
| TVCL[L/h] | 5.93 | 10% |  |
| TVV[L] | 51.9 | 18% |  |
| **Between-subject Variability (BSV)** |  |  |  |
| BSV_CL [%CV] | 41.10% | 9% | 3% |
| **Residual Variability (RV)** |  |  |  |
| Proportional Error [%CV] | 21% | 35% | 33% |

a. BSV calculated as $\sqrt{e^{\omega^{2}}-1}$

**Table S2 Linezolid PopPK parameter estimates from the final model and bootstrap results**

| **Parameter** | | **Final model** | | | | **shrinkage** | | | | | | **Bias (%)** | |
| --- | --- | --- | --- | --- | --- | --- | --- | --- | --- | --- | --- | --- | --- |
|  |  | **Estimate** | | **RSE(%)** | | **2.5th percentile** | | **Median Estimate** | | **97.5^th^ percentile** | |  |  |
| θ_CL_[L/h] | | 3.66 | | 15 | | 2.75 | | 3.72 | | 4.58 | | 1.64 | |
| θ_V_[L] | | 54 | | 17 | | 40.00 | | 54.66 | | 68.08 | | 1.22 | |
| θ_1_ | | 2.18 | | 21 | | 1.33 | | 2.19 | | 3.03 | | 0.46 | |
| I**nter-individual variability** | |  | |  | |  | |  | |  | |  | |
| ω_CL_ [%] | | 36.30 | | 10 | | 27.43 | | 36.36 | | 43.43 | | 0.16 | |
| Residual Variability | |  | |  | |  | |  | |  | |  | |
| σ_pro_(%) | | 19.05 | | 29 | | 13.01 | | 18.30 | | 23.59 | | -3.94 | |

PopPK, population pharmacokinetic; RSE(%), relative standard error; θ_CL_, typical value of apparent clearance; θ_V_, typical value of apparent volume of distribution; θ_1_, allometric value for CrCL as covariate for CL; ω_CL_, square root of inter-individual variance for CL; σ_pro_, residual variability for proportional error.

Bias (%)=(Median Estimate _Bootstrap_ - Estimate _Final model_)/Estimate _Final model_×100%

**Table S3 Comparison of pharamcokinetic parameters of linezolid estimated in this study with those in the literature**

| **Study** | **Subject characteristics** | **Data** | **Structural model** | **Pharmacokinetic parameters and formulas** | **Covariates tested** | **Retained covariates in final model** |
| --- | --- | --- | --- | --- | --- | --- |
| **present study** | **Adult critically ill patients** | **Sparse data from a TDM study** | **1-compartment with first-order elimination** | **CL=3.66+2.18×CrCL/65 L/h V=54 L** | **gender, age, height, weight, white blood cell count (WBC), total bilirubin (TBIL), serum albumin (ALB), alanine aminotransferase (ALT), aspartate aminotransferase (AST), serum creatinine (SCr) and Creatinine clearance (CL_Cr_)** | **CL: WT** |
| Abe et al. 2009 | Adult patients with lower body weight and elderly patients | Rich data from 5 phase II/III studies | 1-compartment with first-order absoption and first-order elimination | Ka=0.583 h^-1^ CL=1.28×(WT/69.5)^1.91^+0.0788×(110−AGE)×(1−AGEj)+0.0788×52×AGEj L/h V= 47×(WT/69.5)^0.903^ L where AGEj=1 if age≥58 years and 0 otherwise | Weight, Age, Gender, Ethnicity | CL: WT, Age V: WT |
| Crass et al. 2019 | Adult patients without renal replacement therapy | Sparse data from a TDM study | 1-compartment with linear elimination | Ka=1.4 h^-1^ CL=3.43+3.49×(BSA-1.89)-0.0242×(AGE-40)×AGEj+1.77×eGFR/80 L/h V=42.90×e^0.901×(BSA-1.89)^ L where AGEj=1 if age＞40 years and 0 otherwise | Weight, Age, Gender, Height, Body surface area (BSA), Serum creatinine, Intensive care unit admission | CL: BSA, Age V: BSA |
| Garcia-Prats et al. 2019 | Pediatric patients with multidrug-resistant tuberculosis (MDR-TB) | Rich data from 2 perspective observational PK studies | 1-compartment with linear elimination | Ka=0.77 h^-1^ CL/F=4.73×(WT/70)^0.75^ L/h V=54.8×(WT/70) L | Weight, Age, Gender, Height, Ethnicity, HIV status, Linezoid administration method | CL: WT V: WT |
| Keel et al. 2011 | Adult patients with cystic fibrosis | Rich data from a PK study | 2-compartment with time-dependent clearance inhibition | F=85.1% Ka=1.91+0.0474×LBW h^-1^ CL=9.54+0.0335×LBW L/h V1=26.8 L V2=17.3 L Q=104 L K_ic_= 0.0005 h^-1^ RCLF=32.1% IC_50_=0.38 mg/L | Weight, Height, Lean body weight (LBW), | Ka: LBW CL: LBW |
| Li et al. 2019 | Pediatric patients | Sparse data from a PK study | 1-compartment with first order elimination | CL=1.31×(lnWT/2.4)^0.83^×(lneGFR/4.89)^0.6^ (L/h) V=4.24×(lnWT/2.4)^0.86^ | Weight, Age, Gender, Height, BSA, Blood urea nitrogen (BUN), Serum creatinine, Uric acid, serum cystain C, Total bilirubin, Alanine aminotransferase, Aspartate aminotransferase, γ-glutamyltranspeptidase, eGFR | CL: WT, eGFR V: WT |
| Luque et al. 2014 | Neurosurgical critically ill patients | Rich data from a PK study | 3-compartment with linear elimination | CL=16.6 L/h V1=43.2 L V2=58 L V_CSF_=0.11 L Q=3.1 L/h Q_CSF_=0.05 L/h | Weight, Age, Gender, Serum creatinine concentration, APACHE score, Use of vasopressors | None |
| Matsumoto et al. 2014 | Adult patients | Sparse data from a PK study | 1-compartment with first order absorption and linear elimination | Ka=0.583 h^-1^ CL=0.0258×CL_Cr_+2.03 L/h V=27.6 L | Weight, Age, Serum creatinine, Creatinine clearance (CL_Cr_), BUN | CL: CL_Cr_ |
| Plock et al. 2007 | 10 healthy volunteers and 24 septic patients | Rich data from a PK study | 2-compartment with parallel linear and Michaelis-Menten elimination | F=100% Ka=1.81 h^-1^ ALAG1=1.27 h CL=11.1 L/h V1=20 L V2=28.9 L Q=75 L/h RCLF=0.764 Kic=0.0019 h^-1^ IC_50_=0.1 mg/L | NA | NA |
| Sasaki et al. 2011 | Adult patients of Japanese | Sparse data from a PK study | 1-compartment with linear elimination | Ka=0.583 h^-1^ CL=2.85×(CL_Cr_/60.9)^0.618^×0.472^CIR^ L/h V=33.6 L | Weight, Age, Serum creatinine, Creatinine clearance (CL_Cr_), Total bilirubin, Liver cirrhosis (CIR) | CL: CL_Cr_, Severe liver cirrhosis V: Weight |
| Soraluce et al. 2020 | Adult critically ill patients including patients subjected to continuous renal replacement therapy | Rich data from a PK study | 2-compartment with linear elimination | CL=2.62+4.35×(CL_Cr_/44)+CL_EC_j L/h V1=16.1 L V2=29.1 L Q=72.3 L/h where CL_EC_ was the extracorporeal clearance for each patients undergoing CRRT calculated as the ratio of linezolid area under the effluent concentration curve to the area under the plasma concentration curve over the dosage interval and mulitply the effluent flow | Weight, Age, Gender, Height, BMI, Serum creatinine, Creatinine clearance (CL_Cr_), Glucose, Hemoglobin, Hematocrit, Albumin, Total proteins, Bilirubin, GPT, GOT, APACHE score, CVVHDF, CVVHD | CL: CL_Cr_ |
| Taubert et al. 2016 | Adult critically ill patients | Rich data from a PK study | 2-compartment with first-order elimination and absorption | CL=7.92×(fibrinogen/12.8)^0.04^×(lactate/1.5)^-0.21^×1.82(if ARDS) L V1=26.55× (WT/74)^1.31^×1.53(if peritonitis) V2=15 L | Weight Height, Peritonitis, Fibrinogen, Antithrombin, Lactate, C-reactive protein, Creatinine clearance (CL_Cr_), ADRS | CL: Fibrinogen, Lactate, ADRS V: Weight, Peritonitis |
| Töpper et al. 2016 | Adult critically ill patients | Sparse data from a PK study | 1-compartment with linear elimination | CL=5.71 L/h V=41.1 L | Weight, Age, eGFR, Albumin, Bilirubin | None |
| Tsuji et al. 2013 | Adult low body weight patients with renal dysfunction | Rich data from a PK study | 1-compartment with linear elimination | CL=0.00327×WT×eGFR^0.428^×HB^0.502^×ALTj L/h V=1.31×WT L Where ALTj=1 if age ≤ 40 and 0.283 otherwise | Weight, Age, Gender, Duration of administration, Serum creatinine, Hemoglobin (HB), Platelets, eGFR, Alanine amino transferase (ALT) | CL: Weight, eGFR, Hemoglobin, ALT V: Weight |
| Tsuji et al. 2017 | Adult patients | Rich data from a PK study | 2-compartment with first order absorption and elimination | F=0.922 ALGA=3.61 h CL=1.86×e^-0.0205×(AGE-69)^+1.44×CL_Cr_/100 L/h V1=22.9×(WT/70) L V2=24.7×(WT/70) L Q=10.9×(WT/70) L/h | Weight, Age, Creatinine clearance (CL_Cr_) | CL: CL_Cr_, Weight V: Weight |
| Wang et al. 2019 | Adult patients with different types of shock | Sparse data from a TDM study | 1-compartment with linear elimination | CL=11.8×(PLT/2000)^0.261^ L/h V=209 L | Sex, Age, Albumin, Globulin, Alanine transaminase (ALT), Aspartate transaminase (AST), Serum creatinine, Urea, Total protein, Total bile acid(TBA), Total bilirubin (TBIL), Hematocrit (HCT), Hemoglobin (HGB), Mean corpuscular hemoglobin (MCH), Mean corpuscular hemoglobin concentration (MCHC), Platelet count (PLT) | CL: PLT |
| Whitehouse et al. 2005 | Adult critically ill patients | Rich data from a PK study | 2-compartment with linear elimination | DI=0.5 h CL=0.0487×WT L/h V1=0.634×WT L V2=240 L Q=7.48 L/h | Weight, Height, Age, Gender, Renal function, Renal replacement therapy, | CL: Weight V: Weight |
| Xie et al. 2019 | Adult obese patients with penumonia | Rich data from a PK study | 2-compartment with linear elimination | CL=7.8×(1-0.0331×(Age-60))×(Weight/70)^0.75^ L/h V1=14.3×(Weight/70) L V2=23.8×(Weight/70) L Q=65.1×(Weight/70)^0.75^ L/h | Weight, Age, Gender, Severity of sepsis episode, Creatinine clearance, Serum albumin, Alanine transaminase, Aspartate Aminotransferase, Total bilirubin | CL: Age, Weight V: Weight |
| Zhang et al. 2016 | Adult healthy volunteers and infected patients of Chinese | Rich data from a PK study | 1-compartment with linear elimination | CL=4.6957+0.0398×(WT-60)-0.0451×(Age-55) L/h V=38.8456+0.7904×(WT-60) L | Weight, Age, Height, Gender, APACHE score, Renal function, Liver function | CL: Age, Weight V: Weight |

**Figure S1**


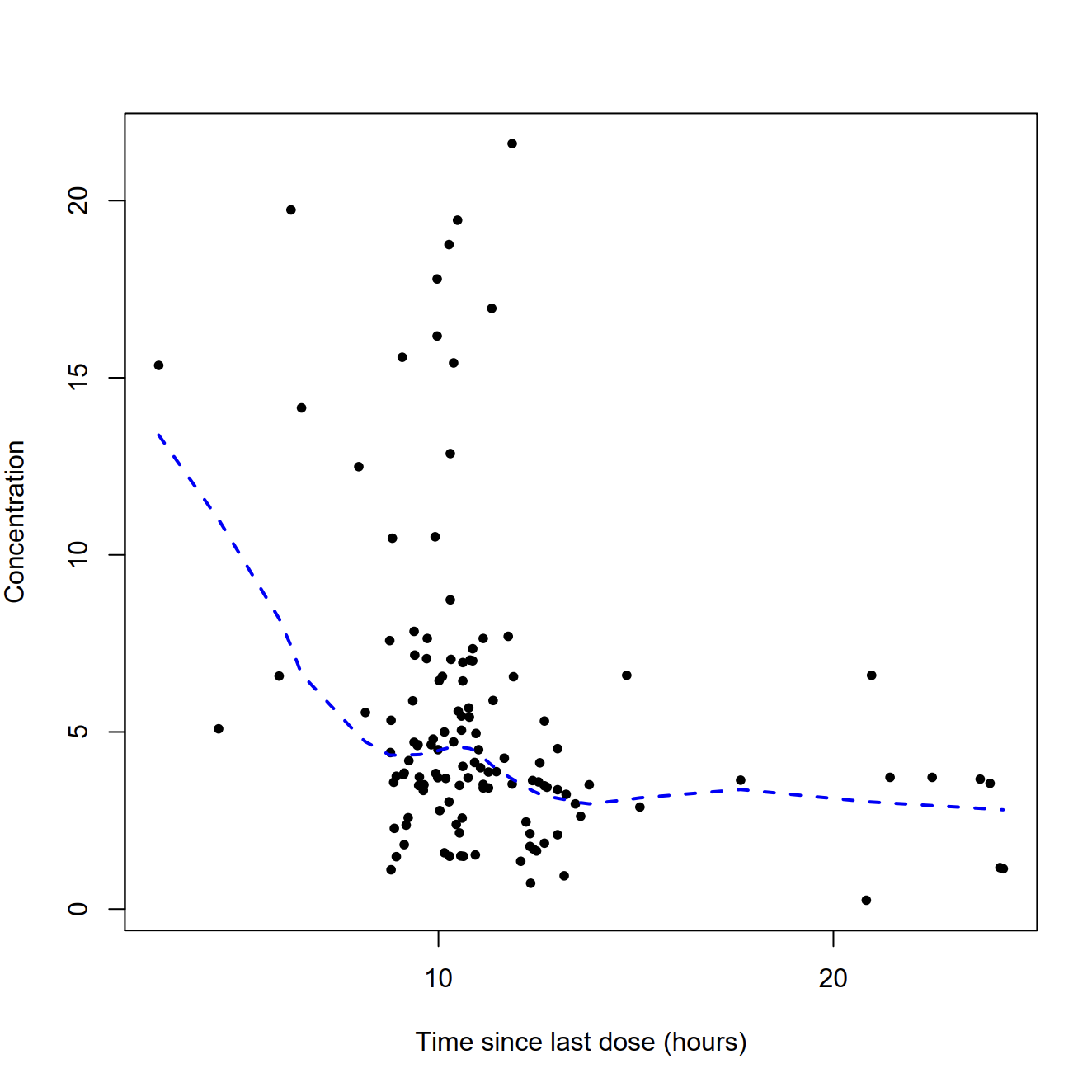


Figure S1. Plasma concentration – time profile of linezolid for the included patients.

**Figure S2**


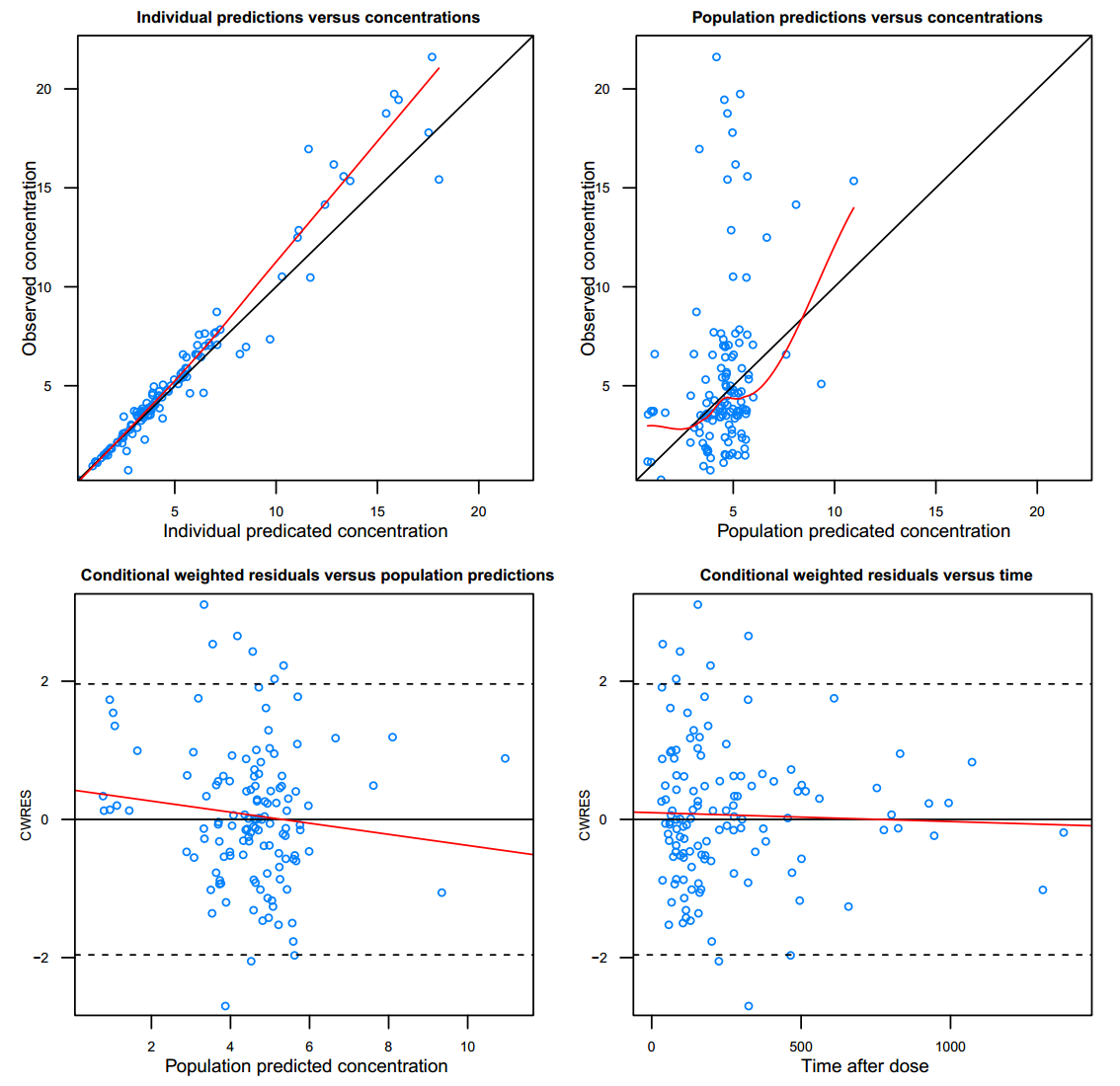


Figure S2. Diagnostic goodness-of-fit plots of the base model. a Observed concentration (DV) vs. individual predicted concentration (IPRED); b DV vs. population predicted concentration (PRED); c conditional weighted residuals (CWRES) vs. PRED; and d CWRES vs. time. The red lines in the upper panel represent loess smooth lines and linear fit lines, respectively.

**Figure S3**


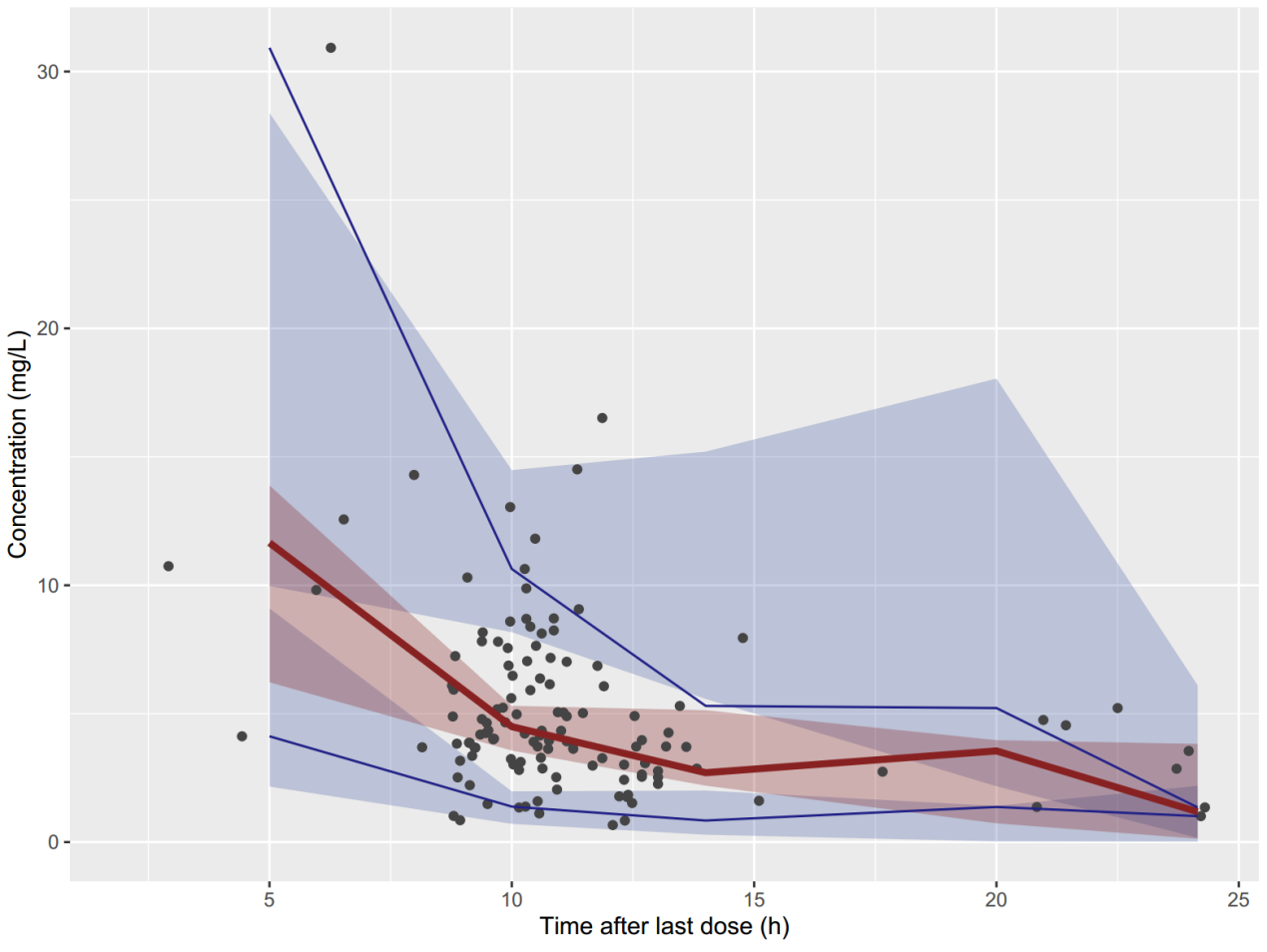


Figure S3. Prediction- and variability-corrected visual predictive check (pvcVPC) plot of the final model. The red solid lines represent the median observed concentration, and the semitransparent red fields represent the simulation-based 90% confidence intervals (CIs) for the median. The observed 5th and 95th percentiles are represented by red dashed lines, and the 90% CIs for the corresponding model predicted percentiles are shown as semitransparent blue fields. The observed concentrations are represented by dark dots.
